# Examining the Interplay of Generalized and Personal Views on Aging on Physical and Mental Health Across 2.5 Years

**DOI:** 10.1093/geroni/igaa057.1969

**Published:** 2020-12-16

**Authors:** Allyson Brothers, Anna Kornadt, Abigail Nehrkorn-Bailey, Hans-Werner Wahl, Manfred Diehl

**Affiliations:** 1 Colorado State University, Fort Collins, Colorado, United States; 2 University of Luxembourg, Esch-sur-Alzette, Diekirch, Luxembourg; 3 University of Heidelberg, Heidelberg, Baden-Wurttemberg, Germany

## Abstract

It remains unknown how distinct types of views on aging (VoA) are related to one another, and to aging outcomes. We used a latent-variable structural equation model to test the hypothesis that generalized views on aging (assessed as Age Stereotypes (AS)) would influence personal views on aging (assessed as Self-Perceptions of Aging (SPA)), which in-turn would influence later physical and mental health. Data came from a longitudinal survey on VoA (N= 537, MageT1 = 64.13, age rangeT1 = 40-98). As expected, SPA mediated the effect of AS on physical (loss-SPA: β = .23, p< .001; gain-SPA: β = .06, p< .001; R2 = .62) and mental health (loss-SPA: β = .13, p< .001; gain-SPA: β = .03, p< .01, ; R2 = .31). Congruent with theoretical assumptions, our findings provide empirical support for a directional pathway by which generalized views on aging affect health outcomes via personal views of aging.

